# Movements that are Both Variable and Optimal

**DOI:** 10.2478/v10078-012-0058-9

**Published:** 2012-10-23

**Authors:** Mark L. Latash

**Affiliations:** 1 The Pennsylvania State University, USA.

**Keywords:** motor control, abundance, variability, optimization, synergy

## Abstract

This brief review addresses two major aspects of the neural control of multi-element systems. First, the principle of abundance suggests that the central nervous system unites elements into synergies (co-variation of elemental variables across trials quantified within the framework of the uncontrolled manifold hypothesis) that stabilize important performance variables. Second, a novel method, analytical inverse optimization, has been introduced to compute cost functions that define averaged across trials involvement of individual elements over a range of values of task-specific performance variables. The two aspects reflect two features of motor coordination: (1) using variable solutions that allow performing secondary tasks and stabilizing performance variables; and (2) selecting combinations of elemental variables that follow an optimization principle. We suggest that the conflict between the two approaches (a single solution vs. families of solutions) is apparent, not real. Natural motor variability may be due to using the same cost function across slightly different initial states; on the other hand, there may be variability in the cost function itself leading to variable solutions that are all optimal with respect to slightly different cost functions. The analysis of motor synergies has revealed specific changes associated with atypical development, healthy aging, neurological disorders, and practice. These have allowed formulating hypotheses on the neurophysiological mechanisms involved in the synergic control of actions.

## Two aspects of synergies: Abundance and optimality

At any level of description, the neuromotor system has more elements (such as joints, digits, muscles, motor units, etc.) than the number of constraints associated with typical tasks. As a result, any task can be performed by a large (infinite) number of combinations of elemental variables. For example, a given location of the tip of the index finger in the external space may be potentially reached with an infinite number of joint configurations; a moment of force in a joint crossed by several muscles can be reached using an infinite number of muscle force combinations; a desired level of muscle activation can be produced by many different subsets of motor units recruited at variable frequencies, etc. Traditionally, this feature has been addressed as redundancy, and N.A. Bernstein formulated one of the main problems of motor control as the problem of motor redundancy (Bernstein, 1967). Motor redundancy is a major factor contributing to what Bernstein called “repetition without repetition”; this phrase implies that repetitive attempts at the same task are accompanied by variable trajectories of elemental variables.

Natural, purposeful human movements have two features that are rarely considered together. On the one hand, motor patterns are variable reflecting two types of variability, state variability and trajectory variability (reviewed in Newell and Corcos, 1993). The former reflects the mentioned excess of elemental variables (those produced by elements) while the latter can be applied even to one-element systems that can show different time profiles while moving from the initial state to a target state.

On the other hand, despite the apparent motor redundancy, human motor patterns show a high degree of consistency across both tasks and persons. Some of those rules, not directly imposed by the task constraints, have been studied extensively. Deviations from such typical patterns are sometimes considered as correlates of various neurological, peripheral, developmental or cognitive disorders (Latash and Anson, 1996). Optimization has been one of the most commonly used approaches to identifying such natural patterns.

The notions of variability and optimality seem contradictory, if not mutually exclusive. Indeed, for a given task, only one combination of elemental variables is optimal. However, “repetition without repetition” suggests that numerous such combinations are realized. Only one of those is optimal with respect to a given objective (cost) function, while others violate the optimality principle to different extents.

The apparent contradiction has its roots in the traditional approach to the problem of motor redundancy. Even the formulation of the problem biases its analysis: The word “problem” implies that it has to be somehow solved – a single solution has to be found; the word “redundancy” implies that one has to eliminate it (cf. Bernstein, 1967). Relatively recently, an alternative approach has been developed that views the design of the human body not as the source of “problems of motor redundancy” but as a “bliss of motor abundance” (Gelfand and Latash, 1998; Latash, 2012). The latter formulation suggests that the design of the human neuromotor system is not a source of problems but a luxury. The purpose of this paper is to provide a review of studies based on the principle of abundance that used two tools for analysis of multi-element behaviors, the framework of the uncontrolled manifold (UCM) hypothesis (Scholz and Schöner, 1999) and the analytical inverse optimization (ANIO, Terekhov et al., 2010).

**Figure 1:**
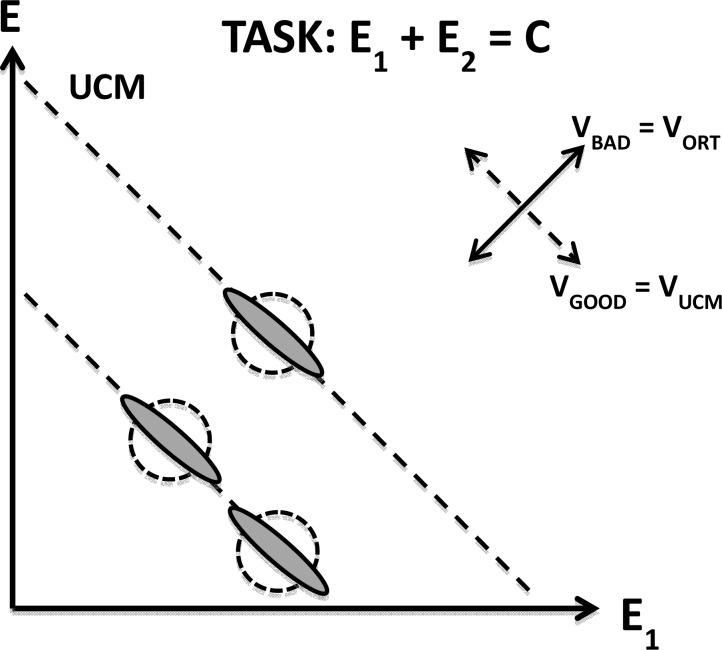
Patterns of performance in an apparently redundant system The task of producing a certain magnitude of the sum of two elemental variables (E_1_+ E_2_= C) may be associated with different data distributions across multiple trials. The distributions may be elongated mostly along the space of solutions (the uncontrolled manifold, UCM, dashed slanted lines) forming ellipses (gray ellipses, synergies stabilizing E_1_+ E_2_) or show a spherical shape (non-synergies). Synergies are defined as distributions with variance along the UCM (V_UCM_or V_GOOD_) larger than orthogonal to the UCM (V_ORT_or V_BAD_). Note that the distributions may differ not only by their shapes but also by the location of their centers.

Figure 1 illustrates possible patterns of performance in a very simple, apparently redundant system: two effectors, for example two index fingers of a person, try to produce a constant combined output: E_1_ + E_2_ = C. There are an infinite number of solutions for the task – points on the dashed slanted lines in Figure 1. These lines are the UCMs for the task corresponding to different values of C; as long as the system sticks to such a line, the controller does not have to interfere to correct the value (E_1_ + E_2_). If a person performs this task many times, individual values of E_1_ and E_2_ are expected to vary across trials. The variability may have a spherical shape (dashed circles in Figure 1) or look like an ellipse (solid ellipses in Figure 1). In the former case, the amount of variance along the UCM (sometimes addressed as “good variance”, V_GOOD_) is equal to that orthogonal to the UCM (V_ORT_ or “bad variance”, V_BAD_). Such distributions have been addressed as non-synergies with respect to the task. In the latter case, if the amount of V_GOOD_ is higher than that of V_BAD_, the result suggests a synergic organization that helps to reduce variance of the performance variable (E_1_ + E_2_) given the variances of the elemental variables. A metric of synergy has been used reflecting the normalized difference between the two variance components, both quantified per dimension in the corresponding sub-spaces: ΔV = (V_GOOD_ – V_BAD_)/V_TOTAL_.

The clouds of data points may be centered about different portions of the UCM. The location of the center of such a distribution reflects the averaged, preferred sharing of the task between the effectors. Both spherical distributions (non-synergy, ΔV = 0) and elliptical distributions (synergies, ΔV > 0) can be centered about different portions of the UCMs. Commonly, the relative sharing does not change with the task magnitude – two distributions are illustrated for both synergic and non-synergic distributions in Figure 1 characterized by different average sharing of C between E_1_ and E_2_. The method of the UCM hypothesis does not address directly possible changes in the sharing. A complementary method has been developed, called analytical inverse optimization (ANIO).

The idea of ANIO is to assume that a certain function is optimized across tasks with different magnitudes of the task parameters and then to reconstruct such a function based on a set of observations. So far, the method has been developed for only some tasks and functions (Terekhov et al., 2010; Terekhov and Zatsiorsky, 2011); in particular, it has been applied successfully to tasks of simultaneous total force and moment of force production by the four fingers of the hand.

ANIO has an advantage over more traditional (direct) optimization methods that involve guessing a cost function, for example based on some reasonable mechanical, physiological, or psychological considerations supported by the researcher’s intuition. ANIO does not assume a cost function but computes it. Further, the cost function is used to predict performance over the same set of task parameters. Comparisons of the performance of such computed cost functions with a set of traditionally used ones have shown advantage of the ANIO method (Niu et al., 2012).

**Figure 2:**
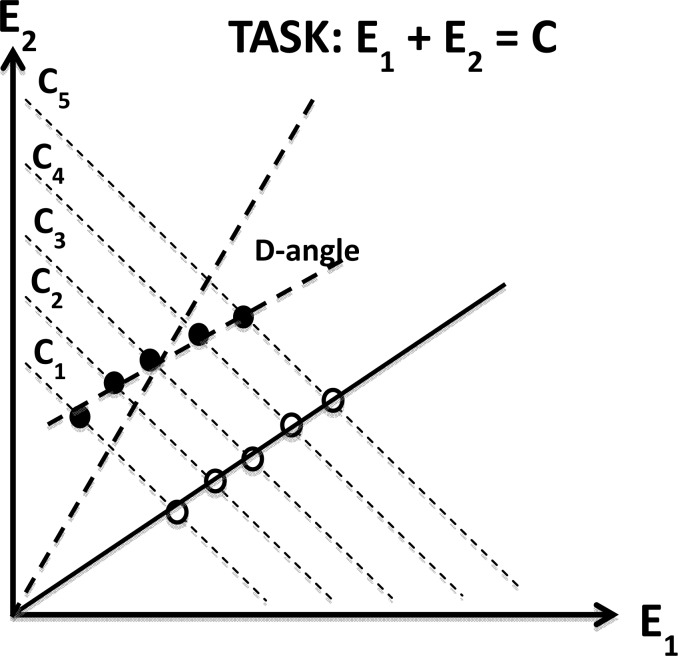
Two sets of data points for different values of C *If the same task, as in* Figure 1, is performed over a range of values of C, the data points may consistently follow a sharing pattern (open circles) or not (black circles). In the former case, it is possible to claim that the performance follows consistently an optimization principle, while in the latter case there is a large angle (D-angle) between the space of the data points and the space of optimal solutions computed using Analytical Inverse Optimization (ANIO).

Figure 2 illustrates two sets of data points for different values of C. One of the sets (open circles) consistently follows a sharing pattern over the whole range of C values. One can say, that this data set shows high consistency in following an optimization principle (solid line). The other set (filled circles), does not follows a consistent sharing pattern, and individual observations show much larger deviations from the line of “optimal performance” (dashed line). Different metrics have been used to describe the consistency of sticking to an optimization principle. One of them is the dihedral angle (D-angle) between the sub-space of actual data points and a sub-space of computed values based on the cost function; smaller D-angle magnitudes correspond to higher consistency in following an optimization principle.

Analyses similar to the ones illustrated in Figures 1 and 2 can be performed for systems with more than two elements and different tasks. The main outcome variables of such analyses (for example, ΔV and D-angle) describe properties of the shape and location of the experimentally observed data point distributions respectively.

The UCM method is already about 13 years old. Over this time, the method has been applied to analysis of a variety of actions performed in a variety of conditions by a variety of populations (reviewed in Latash et al., 2002b; 2007; 2010). Overall, the method has proven its sensitivity to such important factors as practice, neurological or developmental disorder, aging, fatigue, etc. Here follow a few examples of the application of the UCM-based analysis of synergies (with or without the ANIO method).

## Benefits of synergies

From the definition of a synergy illustrated in Figure 1 it is clear that variability of performance is defined only by the V_BAD_ component of variance. By definition, V_GOOD_ has no direct effect on performance. Why would the central nervous system facilitate comparatively large amounts of V_GOOD_ resulting in high synergy indices?

**Figure 3:**
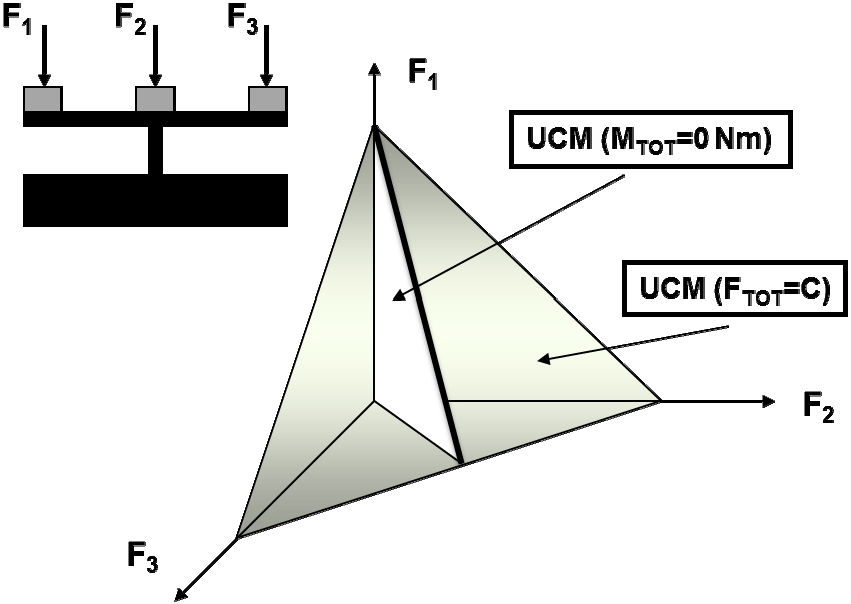
3D space of finger forces and the UCMs for the task of producing a certain value of the total force and a zero total moment of force while pressing with three fingers One of the advantages of having large amounts of “good variance” is the possibility to perform secondary tasks. The plot shows two uncontrolled manifolds (UCMs). The gray triangle corresponds to the UCM for a fixed total force, while the white triangle corresponds to the UCM for zero total moment of force in the configuration shown in the insert. The thick black line is the space of solutions for both tasks, F_TOT_= C and M_TOT_= 0.

Recent studies suggested that there may be two benefits from using high V_GOOD_. First, large amounts of V_GOOD_ allow the controller to use this sub-space to perform secondary tasks without interfering with the original task (Zhang et al., 2008). For example, Figure 3 shows a 3D space of finger forces and the UCM for the task of producing a certain value of the total force while pressing with three fingers (the gray triangle). If, in addition to performing the task, the person has to balance the frame with the sensors on a narrow pivot (see the insert), a secondary task emerges that requires accurate production of the moment of force. A large amount of V_GOOD_ allows the subject of this experiment to select a sub-space within the first UCM that satisfies the second task (thick line). In more intuitive terms, if one walks down the hallway with a mug of coffee in the hand, large V_GOOD_ allows to open a door by pressing on the handle with the elbow of the same hand and not spilling the coffee.

Another important benefit of having large amounts of V_GOOD_ is to ensure stability of performance in the presence of unavoidable intrinsic (“noise”) and extrinsic perturbations. One of the first studies on the kinematic synergies during quick-draw pistol shooting documented strong synergies (large V_GOOD_) in such tasks (Scholz et al., 2000). When the subjects were asked, without any practice, to perform the task with a rubber band crossing the elbow joint, most of them hit the target accurately at the first attempt. This was possible only because the unexpected (and complex!) effects of the perturbation associated with the rubber band action were channeled mostly into the UCM for this task. A later study quantified the amount of joint configuration deviation introduced by a similar elastic band during quick reaching movements in the self-motion and range-motion sub-spaces (Mattos et al., 2011). Most of the deviation was within the self-motion space (≈UCM) confirming the idea that high V_GOOD_ and the ability to channel effects of perturbations into a sub-space that has no effect on an important performance variable are related to each other.

As the last couple of examples suggest, the notion of synergies is tightly linked to the issue of movement stability. Performing a task involving a redundant set of effectors may be viewed as associated with setting time profiles of two types of neural variables. One of them defines trajectories in the space of elemental variables that can be seen in averaged across trials patterns. The other defines patterns of co-variation among the elemental variables across trials. Several recent studies have shown that patterns of co-variation can change independently of the averaged across trials performance (Olafsdottir et al., 2005; Shim et al., 2005b; 2007; Klous et al., 2011). For example, when a person performs a steady-state task and then a quick change in the performance variable, the synergy index shows a drop 150–200 ms prior to the action initiation. These, so-called anticipatory synergy adjustments (ASAs) represent an example of changing co-variation patterns to attenuate synergies stabilizing the performance variable; otherwise, the person would be fighting his or her own synergies.

In other studies, subjects performed a quick reaching movement in conditions of possible changes in the target location (Freitas and Scholz, 2009). In some series, the target could jump to a new location after the movement had been initiated. Compared to similar series when the target never changed location, in the series when target could jump the synergy index was decreased, even when the index was computed over trials when the target stayed stationary. Taken together, these studies provide support for a direct link between the synergy index and stability of performance.

## Effects of age and neurological disorder

Synergies show major changes with atypical development, healthy aging, and neurological disorder. These studies point at potential clinical importance of studying and quantifying synergies and also allow formulating hypotheses on the neurophysiological mechanisms involved in the synergic control of action.

Two findings have suggested a novel hypothesis on neurophysiological changes with aging (Shinohara et al., 2004; Kapur et al., 2010). First, elderly persons show lower indices of enslaving – that is, better indices of finger individuation! – as compared to younger persons. This may be the only index of motor performance that shows a benefit of aging. Second, indices of synergies during a variety of tasks are reduced in the elderly. Note that any task can be performed by either specifying involvement of individual elements or uniting elements into synergies and using a smaller number of neural variables. According to the “back-to-elements” hypothesis (Kapur et al., 2010), aging reverses the natural process of the formation of synergies relevant to everyday tasks. When a child grows up, the neural control of everyday movements is shifted from element-based to synergic. With advanced age, the loss of neurons at many levels of the neural hierarchy leads to destruction of some of the neural connections involved in the synergic control, and the central nervous system switches back to the element-based control. In some sense, with advanced age, people become younger.

Significant changes in synergies have been documented in patients with subcortical disorders such as Parkinson’s disease and olivoponto-cerebellar atrophy (OPCA) (Park et al., 2012). These changes include several components. First, the synergy indices during steady-state tasks drop. Second, ASAs in preparation to a quick action are delayed and decreased in magnitude. Third, ANIO reveals less consistent use of optimal solutions for wide ranges of tasks variables (this later finding has also been seen – to a lesser degree – in healthy elderly). These observations point at important roles of the basal ganglia and the cerebellum in the formation of synergies and their adjustments to specific tasks. This hypothesis is also supported by the observations of impaired multi-finger synergies in Down syndrome (Latash et al., 2002a), which is associated with cerebellar abnormalities. It is interesting that studies of multi-joint reaching in stroke survivors showed minor differences in the synergy indices between the more impaired (contralesional) and less impaired (ipsilesional) arms (Reisman and Scholz, 2003). Taken together, the studies point at a central role of subcortical structures in the synergy formation.

## Changes in synergies with practice

Motor practice has traditionally been viewed as a staged process associated with elimination and later release of redundant degrees-of-freedom, DOFs (Bernstein, 1996; Newell, 1991). Note that this view was developed within the traditional approach to the “problem of motor redundancy”.

Recent studies using the framework of the UCM hypothesis have documented no changes in the number of DOFs but two stages in how these DOFs are coordinated to stabilize importance performance variables (Domkin et al., 2002; Latash et al., 2003; Kang et al., 2004). If a subject faces a completely novel motor task, no synergies stabilizing performance may be seen. With practice, such synergies emerge, reflected in proportionally higher V_GOOD_ as compared to V_BAD_, and strengthen (the synergy index, ΔV grows). Typically, both variance indices show a drop, but V_BAD_ drops faster. If the subject continues to practice, no further decline in V_BAD_ may be possible due to a floor effect. Meanwhile, V_GOOD_ continues to drop, possibly reflecting search for more optimal solutions of the task based on factors that may be beyond the explicit task formulation. As a result, during this second stage, the synergy index drops. Experiments with transcranial magnetic stimulation (TMS) have shown that practice-related changes in synergies are reflected in changes in the excitability of corticospinal pathways as well as in inter-hemispheric inhibitory effects when the task involved both upper extremities (Latash et al., 2003; Shim et al., 2005a).

A recent study has been designed to encourage synergy formation and strengthening (Wu, Pazin, Zatsiorsky, Latash unpublished). In that study, two important features were implemented. First, the subjects never repeated the same task but were forced to perform a family of tasks created using variable parameters to adjust template portions of the task. Second, task stability was decreased as the subjects improved their performance using software tools without changing the actual task mechanics and the type of feedback the subjects received. The first results are encouraging. Subjects who practice a redundant task in such conditions showed a drop in V_BAD_ and an increase in V_GOOD_ resulting in an increase in the synergy index and an increase (!) in the total variance in the space of elemental variables. Subjects who practiced one element at a time showed no changes in the synergy index, both V_BAD_ and V_GOOD_ dropped proportionally. These results show that synergies can indeed be trained by properly designed tasks; this promises direct application to clinical rehabilitation of patients with disordered synergies.

## Variability of optimal behavior vs. optimality of variable behavior

The idea of finding a single optimal solution for a multi-element task seems to leave little space for motor variability. This is true, however, only if several consecutive attempts at a task are performed in perfectly reproducible conditions. Obviously, this is impossible to achieve. One interpretation of the across-trials variability is based on the notion of neuromotor noise (Harris and Wolpert, 1998) that produces deviations from a single optimal solution. However, such noise is not expected to show task-specific co-variation among its contributions to the outputs of elements: it is expected to lead to equal contributions to V_GOOD_ and V_BAD_. Experiments show, however, that the across-trials variance is mostly confined to the UCM. These findings speak against attributing the observations to neuromotor noise. Based on the observations of substantial variability across trials with the same values of the task constraints (Park et al., 2010), it is possible to conclude thatoptimality of the observed finger force patterns is not absolute. It may depend on a particular initial state of the system when the task is performed, for example on excitability of relevant neuronal pools, as well on small variations in the external conditions. Hence, each trial is performed starting from a unique state, and optimal solutions may vary across such states.

The patterns of variance quantified with the help of the UCM hypothesis show that, across a variety of tasks, substantial amounts of variability are present in the space of elemental variables that has no effect on important performance variables (V_GOOD_), while variability that affects such variables (V_BAD_) is kept low (reviewed in Latash et al., 2007; Latash, 2008; 2010). The range of deviations along the UCM is, however, limited. For example, in the earlier example illustrated in Figure 1, the values E_1_=0; E_2_=F_TOT_ are never used. Hence, there is a factor that limits the variability range even along its “good” directions. It seems reasonable to assume that this factor reflects an unknown optimization process. So, two potentially independent features of data distributions are likely to be defined by the two principles, optimality and structured variance. The centers of the observed data distributions correspond to average sharing patterns among the effectors reflecting an optimality criterion. The shape of the distributions indicates desired stability properties of the system in producing the required value of performance variable(s) reflecting the relative amounts of “good” and “bad” variance.

Studies of motor synergies promise insights into the neural organization of motor coordination and direct applications to such fields as motor rehabilitation and athletics. This is a very young field with a lot of challenges and white spots. Join the field - it is fun.
